# Supplementary material for: Comparative Transcriptome Analysis of Two Aegilops tauschii with Contrasting Drought Tolerance by RNA-Seq
Source: Int J Mol Sci. 2020 May 19;21(10):3595. doi: 10.3390/ijms21103595 (PMC7279474; doi:10.3390/ijms21103595)
Supplement: Supplementary file 1 [file ijms-21-03595-s001.zip › supplementary/ijms-666666-supplementary figures.pdf]

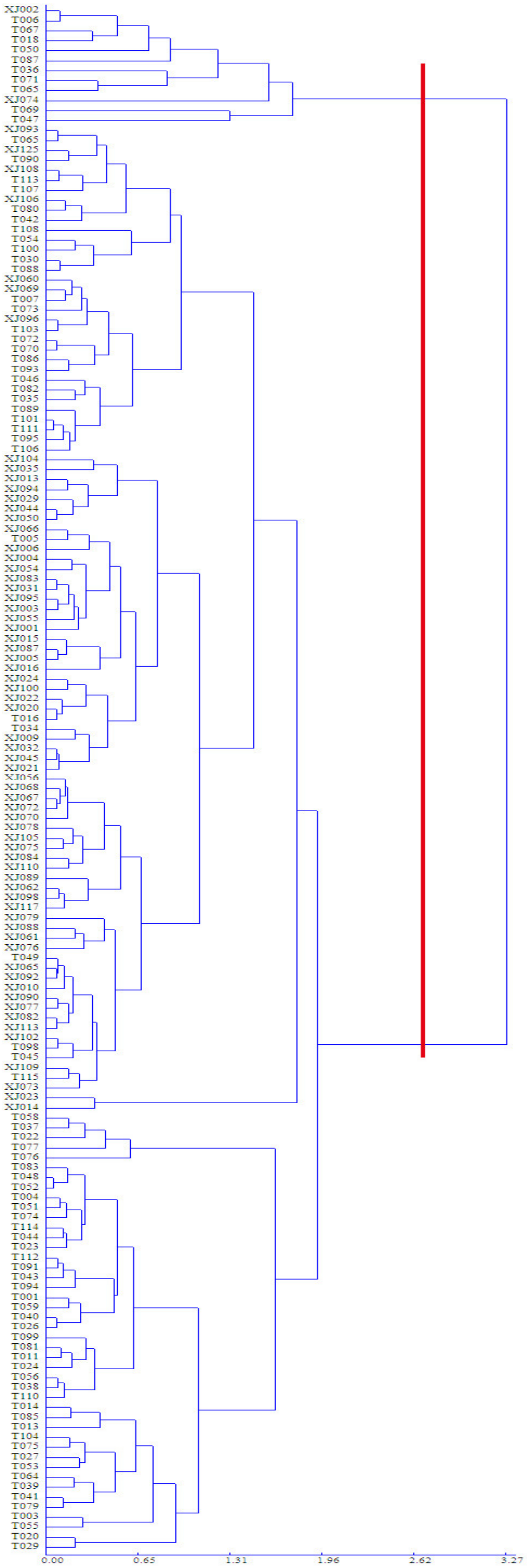

Figure S1 Cluster tree of coleoptile lengths from 155 *Ae. tauschii* accessions acquired through the method of unweighted pairgroup method with arithmetic means (UPGMA) according to Euclidean distances.

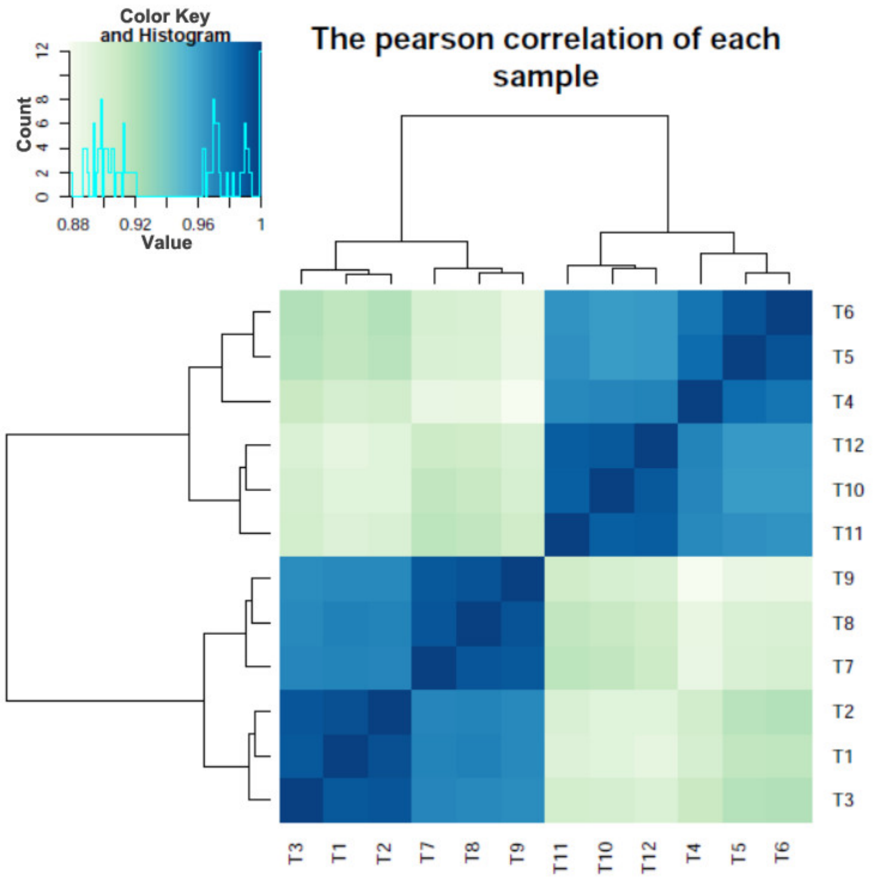

Figure. S2 Pearson correlation coefficients among 3 biological replications from XJ002 and XJ098 under the control and drought stresses. The numbers in the scale bar stand for correlation coefficients. XJ002-control: T1, T2, T3; XJ002-treatment: T4, T5, T6; XJ098-control: T7, T8, T9; XJ098- treatment: T10, T11, T12.

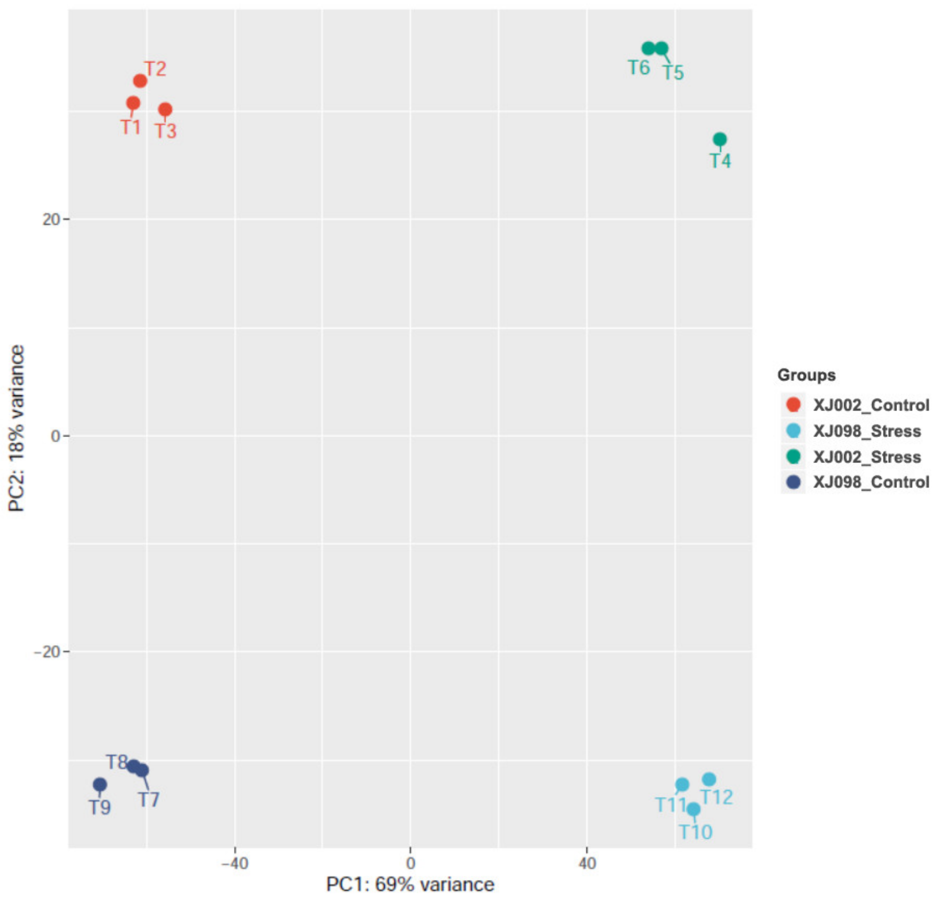

Figure S3 Principal component analysis among 3 biological replications from XJ002 and XJ098 under the control and drought stresses.

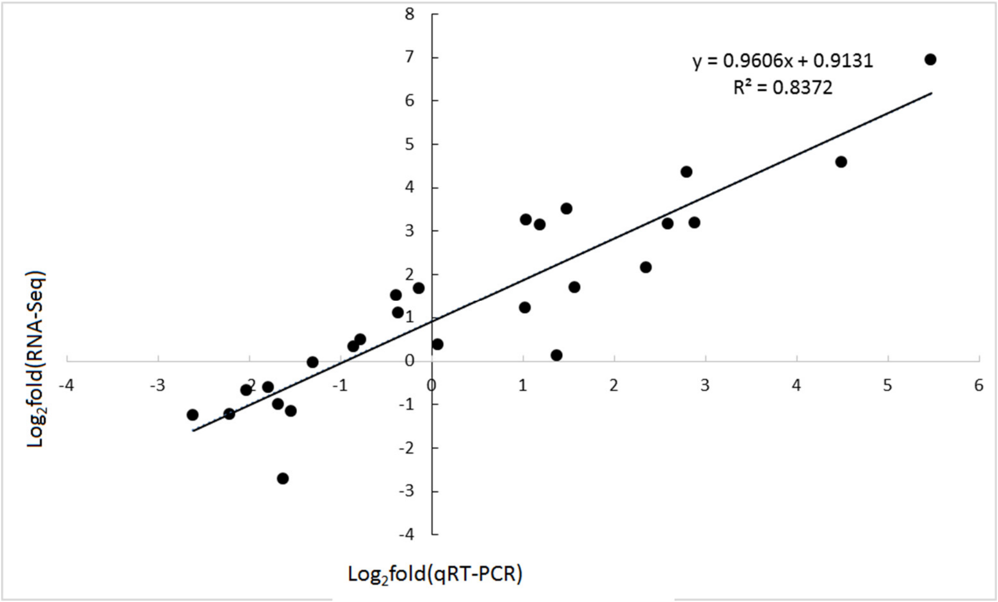

Figure S4 Reliability of DEGs validated by RNA-Seq and quantitative real-time PCR.
